# O.1.1-4 The relationships of mental well-being with physical activity during the COVID-19 in older adults

**DOI:** 10.1093/eurpub/ckad133.080

**Published:** 2023-09-11

**Authors:** Tiina Savikangas, Tiia Kekäläinen, Anna Tirkkonen, Sarianna Sipilä, Katja Kokko

**Affiliations:** Faculty of Sport and Health Sciences and Gerontology Research Center, University of Jyväskylä, Finland; Faculty of Sport and Health Sciences and Gerontology Research Center, University of Jyväskylä, Finland; Faculty of Sport and Health Sciences and Gerontology Research Center, University of Jyväskylä, Finland; Faculty of Sport and Health Sciences, University of Jyväskylä, Finland; tiina.m.savikangas@jyu.fi; Faculty of Sport and Health Sciences and Gerontology Research Center, University of Jyväskylä, Finland

## Abstract

**Purpose:**

Negative mental well-being may hinder, and positive mental well-being facilitate physical activity (PA) when one’s daily routines are compromised. COVID-19 posed challenges to both mental well-being and PA, in Finland especially for those over 70, who faced the strictest restrictions. Therefore, the aim was to investigate the relationships of mental well-being with PA during the COVID-19 in two cohorts of different ages.

**Methods:**

Data came from two population-based studies. Participants of the longitudinal TRAILS study (N = 162, 58% women, 60-61 years) were representative of their age cohort. Participants of the randomized controlled trial PASSWORD (N = 272, 60% women, 72-88 years) had attended a year-long multicomponent physical training intervention during 2017-2019. Self-reported changes in PA (increased vs. no change/decreased; decreased vs. no change/increased) and PA frequency (1-7; from “not at all” to “approximately daily”) during the COVID-19 were collected from April 2020 to June 2020 (PASSWORD) or July 2021 (TRAILS). Positive mental well-being was assessed by positive affect from the International Positive and Negative Affect Schedule Short Form (I-PANAS-SF, score 1-5). Negative mental well-being indicators were negative affect (I-PANAS-SF, score 1-5) and depressive symptoms (TRAILS: General Behavioral Inventory, score 0-3; PASSWORD: Geriatric Depression Scale, score 0-15). Relationships between mental well-being and PA were analyzed using logistic and linear regression models, adjusted by sex and, in PASSWORD, age.

**Results:**

Positive affect was positively associated with increased PA (odds ratio (OR)=1.751-2.661, p = 0.013-0.034) and PA frequency (B = 0.489-0.550, p < 0.001-0.009) in both studies, and inversely with decreased PA in the PASSWORD (OR = 0.633, p = 0.024). Higher negative affect and depressive symptoms were associated with decreased PA in the PASSWORD (OR = 2.134, p = 0.004 and OR = 1.310, p < 0.001, respectively). Additionally, depressive symptoms were associated with lower PA frequency in both studies (TRAILS: B=-0.876, p = 0.004; PASSWORD: B=-0.105, p = 0.002).

**Conclusions:**

Positive mental well-being was consistently and positively associated with PA during COVID-19 in older adults. Higher negative mental well-being was more clearly associated with poor PA behaviors in the older cohort facing stronger restrictions. Supporting positive mental well-being may be as important as reducing negative mental well-being to facilitate physically active lifestyle among older adults during exceptional circumstances.

**Funding:**

The Academy of Finland (323541; 296843).

